# Fano Resonance in a MIM Waveguide with Two Triangle Stubs Coupled with a Split-Ring Nanocavity for Sensing Application

**DOI:** 10.3390/s19224972

**Published:** 2019-11-15

**Authors:** Xiaoyu Yang, Ertian Hua, Mengmeng Wang, Yifei Wang, Feng Wen, Shubin Yan

**Affiliations:** 1 School of Instrument and Electronics, North University of China, Taiyuan 030051, China; 18734146697@163.com (X.Y.); wmm15534033039@163.com (M.W.); 18435132117@163.com (Y.W.); nucwenfeng@163.com (F.W.); 2 School of Electrical Engineering, Zhejiang University of Water Resources and Electric Power, Hangzhou 310018, China; het@zjut.edu.cn

**Keywords:** refractive index sensor, Fano resonance, metal-insulator-metal waveguide, finite element method

## Abstract

Herein, a compact refractive index nanosensor comprising a metal- insulator- metal (MIM) waveguide with symmetric two triangle stubs coupled with a circular split-ring resonance cavity (CSRRC) is theoretically presented. An analysis of the propagation characteristics of the designed structure is discussed employing the finite element method (FEM). The calculation results revealed that a Fano resonance outline emerged, which results from an interaction between the continuous broadband state of the waveguide with two symmetric triangle stubs and the discrete narrowband state of the CSRRC. The influence of geometric parameters on sensing properties was studied in detail. The maximum sensitivity reached 1500 nm/RIU with a high figure of merit of 65.2. The presented structure has great applications for on-chip plasmonic nanosensors.

## 1. Introduction

Surface plasmon polaritons (SPPs) are transverse electromagnetic waves, originating from incident photons coupled with free electrons on the surface of the metal [1,2]. The electric field intensity of SPPs is tightly bound along the metal–dielectric interface and decays significantly in the orientation vertical to the interface [3,4]. SPPs break the classical diffraction limit of light and can realize the optical manipulation within sub-wavelength scales [5,6]; hence, the application of SPPs is extensive [7,8]. There are various SPPs-based waveguides, for instance, stripe waveguides, semiconductor–insulator–semiconductor (SIS) waveguides, metal-insulator-metal (MIM) waveguides, and trench waveguides. Among these, the MIM waveguide has better properties, for instance, stronger confinement of light, shorter propagation length, low band loss, smaller mode size, and low fabrication cost [9,10]. To date, a number of the optical devices of the MIM waveguide structure have been extensively studied, for example, nanosensors [11,12,13,14], demultiplexers [15], splitters [16,17], filters [18,19,20], and optical switching [21,22].

Special optical effects have been found in the SPP waveguide system, such as plasmonically induced reflection (PIR) [23], Fano resonance [24,25,26], and electromagnetically induced transparency (EIT) [27,28]. Differing from the traditional symmetric Lorentzian lineshape, Fano resonance produces a sharp and asymmetric spectral lineshape and exhibits a narrower full width at half maximum (FWHM) [29]. Additionally, Fano resonance is greatly susceptible to the variation of structural parameters and medium environment. Hence, combining Fano resonance and MIM structures can achieve high sensitivity with an excellent figure of merit (FOM). Heretofore, numerous refractive-index nanosensor systems based on Fano resonance have been studied. Wang et al. [30] proposed a Fano system of the plasmonic waveguide coupled with a T analogs cavity, with a sensitivity of 680 nm/RIU. Zhao et al. [11] reported a sensor based on MIM structure, that comprised a ring cavity and an F-P cavity, and its sensitivity could reach 718 nm/RIU. Qiao et al. [14] designed a nanosensor composed of a baffle coupled with an M-type cavity, which could attain a sensitivity of 780 nm/RIU. Compared with fiber sensors, plasmonic sensors [31,32] have many advantages; however, they have a lower sensitivity. Therefore, it is crucial to improve the sensitivity of plasmonic sensors [33].

In this paper, a novel refractive index nanosensor is theoretically presented, which consists of a MIM waveguide with two symmetric triangle stubs coupled with a circular split-ring resonance cavity (CSRRC). The transmission spectra and the H_Z_ field distributions were numerically demonstrated employing the finite element method (FEM). The spectra of the Fano resonance are readily affected by structural parameters of the proposed system. Therefore, the effects of the geometric parameters on the transmission properties were studied. These geometric parameters include the outer radius of CSRRC, the distance between two triangle stubs, the height of the triangle stub, the split size of CSRRC, and the coupling distance.

## 2. Structure Model and Analytical Method

**Figure 1:**
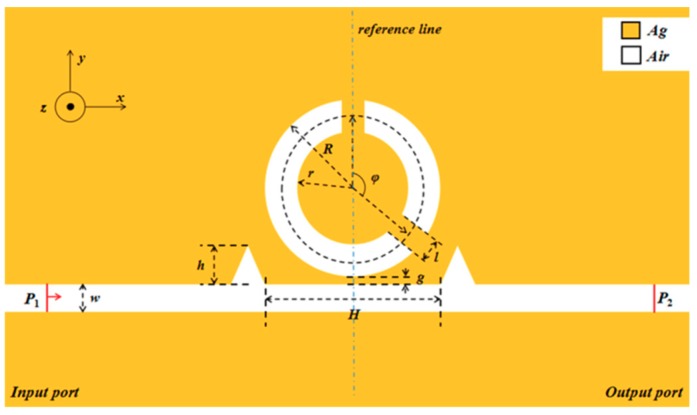
2D schematic of a waveguide with two symmetric triangle stubs coupled with a circular split-ring resonance cavity (CSRRC).

The schematic of the designed sensor is plotted in Figure 1. The structure comprised a MIM waveguide with two symmetric triangle stubs and a CSRRC. Since the thickness of the proposed structure was significantly longer than the light wavelength, the 2D model could be used to approximate the 3D model. As a result, the calculation was simplified and the problem of the computer workstation limitation was fixed. The white and yellow areas in the figure, respectively, represent air and silver. The relative dielectric constant of air is 1 (i.e., $\varepsilon_{d}=1$). The relative dielectric constant of silver is defined by the Debye–Drude dispersion model [34]:

(1) $$\varepsilon (\omega )=\varepsilon_{\infty }+\frac{\varepsilon_{s}-\varepsilon_{\infty }}{1+i\tau \omega }+\frac{\sigma }{i\omega \varepsilon_{0}}$$

where $\varepsilon_{\infty }=3.8344$ denotes the relative permittivity of infinite frequency, $\varepsilon_{s}=-9530.5$ denotes the static permittivity, the relaxation time is taken as τ=7.35× 10^−15^ s, and the conductivity of silver is taken as σ=1.1486× 10^7^ S/m.

The two triangular stubs are symmetric about the reference line. The height of the two triangle stubs is *h*. The distance between the two symmetric triangle stubs is *H*. Two notches are made in the unbroken ring to obtain the CSRRC structure. *R* and *r*, respectively, express the outer and inner radii of the CSRRC. The length of the CSRRC split is *l* and the angle between the two splits of the CSRRC is defined as *φ*. The coupling distance is described by *g* and *w* = 50 nm denotes the width of the CSRRC, the two triangle stubs and the MIM waveguide, which is remarkably shorter than the wavelength of the incident light. Hence, only the fundamental transverse magnetic (TM_0_) mode exists and propagates in the structure [35], which can support SPP waves.

A geometric model of this sensor system was built by employing COMSOL Multiphysics 5.3a [36]. Then, ultra-fine meshing was chosen to ensure the accuracy of calculations. The perfect matched layer (PMLs) was chosen to be the absorbing boundary condition of the designed system, which could greatly absorb the waves emitted from the inside of the structure and prevent all reflected waves from entering the interior of the structure. The input port and output port were marked as P_1_ and P_2_, respectively.

## 3. Simulations and Results

**Figure 2:**
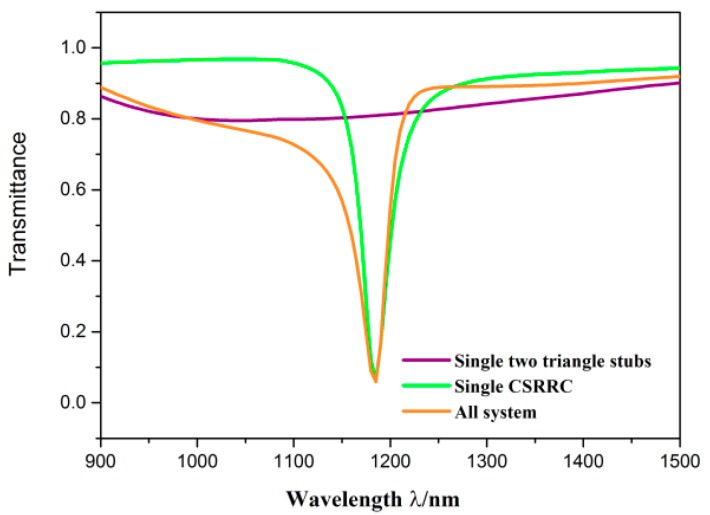
Transmission spectra of the single two symmetric triangle stubs (purple line), the single CSRRC (green line), and the sensor system (orange line).

For a clear understanding of the propagation characteristics of the designed structures, the structural parameters were set as follows: *R* = 130 nm, *l* = 30 nm, *φ* = 135°, *H* = 360 nm, *h* = 80 nm, *g* = 10 nm. The whole system was compared with the single CSRRC structure and the single two symmetric triangle stubs structure, which is depicted in Figure 2. The purple, green and orange solid lines denote transmission spectra of the single two symmetric triangle stubs structure, the single CSRRC structure and the whole system, respectively. It can be observed that the transmission spectrum of the whole system had an obvious Fano resonance, which is characterized by an asymmetrical sharp shape. This phenomenon was aroused by the interaction of the continuous broadband state and the discrete narrowband state [37,38]. The transmission spectrum of the single two symmetric triangle stubs structure has a positive slope, and its entire curve has very similar relatively high transmittance. Thus, it is considered as the continuous broadband state. The transmission spectrum of the single CSRRC structure was similar to the Lorentz shape, which is deemed as the discrete narrowband state.

**Figure 3:**
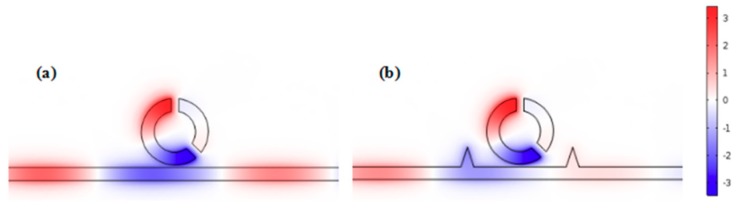
The H_Z_ field distribution at the resonance dip of (**a**) the single CSRRC structure at *λ* = 1185 nm (**b**) the whole system at *λ* = 1185 nm.

To understand the internal mechanism of Fano resonance more clearly, the normalized H_Z_ field distributions of the single CSRRC structure and the whole system at the resonance dip (*λ* = 1185 nm) were studied. They are depicted in Figure 3a,b, respectively. From Figure 3b, it can be seen that the normalized H_Z_ field distribution was only in the left part of the waveguide and the long part of the CSRRC, and a relatively strong resonance was formed in the CSRRC. In addition, the upper and bottom areas of the long part of the CSRRC were out of phase. However, there was normalized H_Z_ field distribution in the right part of the waveguide in Figure 3a. The other area of the normalized H_Z_ field distribution of the single CSRRC structure was similar to that of the whole system. It is to be noted that a stronger resonance exists in the CSRRC when the normal waveguide adds two symmetric triangle stubs, which can promote the formation of Fano resonance. Then, when the strong resonance is excited, the SPPs are almost confined within the CSRRC, which leads to a low transmittance at the dip. It is obvious in Figure 3b that the SPPs are, directly and indirectly, coupled to the waveguide and the CSRRC, respectively. Thus, they can be regarded as the continuous broadband state and the discrete narrowband state, respectively.

**Figure 4:**
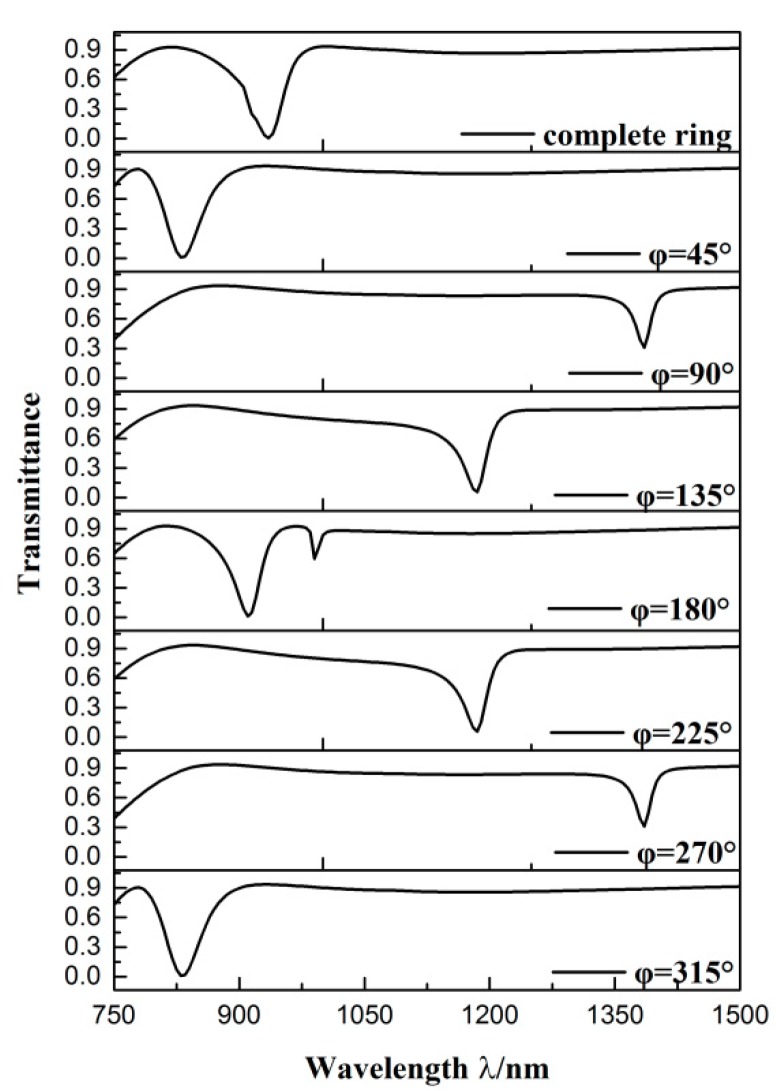
Transmission spectra of the different structures.

We then studied the eight similar structures, whose parameters were all the same as those in Figure 3, except the angle between the two splits of the CSRRC *φ*. Among them, seven structures included two splits, while one structure contained a complete ring as the comparison. We calculated the transmission spectra of the different structures, whose angle between the two splits was 45°, 90°, 135°, 180°, 225°, 270° and 315°, respectively. The transmission spectrum of the complete ring structure was also considered. As shown in Figure 4, there were differences between the transmittance spectra of these eight structures. As for the complete ring, *φ* = 45° and the *φ* = 315° side-coupled CSRRC structures, their transmittance spectra were the Lorentz shape curve, which had ultra-low transmittance at their dip (the complete ring was at *λ* = 935 nm while the other two structures were both at *λ* = 830 nm) and a broad FWHM. As for the *φ* = 90° and *φ* = 270° side-coupled CSRRC structures, their FMHW was relatively narrow, but they had high transmittance at the dip (*λ* = 1385 nm), which leads to difficulty in detecting signals. As for the *φ* = 135° and *φ* = 225° side-coupled CSRRC structures, as we analyzed previously, they showed a Fano resonance phenomenon and thus, had ultra-low transmittance at their dip (*λ* = 1185 nm) and relatively narrow FWHM. The ultra-low transmittance can lead to a larger extinction ratio, and the relatively narrow FWHM can lead to a high figure of merit (FOM) and better sensing resolution. As for the *φ* = 180° side-coupled CSRRC structure, there were two dips (one at 910 nm and the other at 990 nm) in the transmittance spectrum. The first dip (*λ* = 910 nm) had a relatively broad FWHM and low transmittance at the dip, while the second dip (*λ* = 990 nm) had a narrower FWHM and very high transmittance at the dip. In addition, the distance between these two dips was very close, thus they would interfere with each other’s detection. From the above comparison, it can be concluded that proper destruction of the symmetry of the structure can support Fano resonance, which has better sensing properties of high sensitivity alongside a better FOM. It was found that the transmission spectra of the *φ* = 45° and *φ* = 315° side-coupled CSRRC structure, the *φ* = 90° and *φ* = 270° side-coupled CSRRC structure, the *φ* = 135° and the *φ* = 225° side-coupled CSRRC structure were the same. This is because the complete ring structure was symmetrical about the reference line.

**Figure 5:**
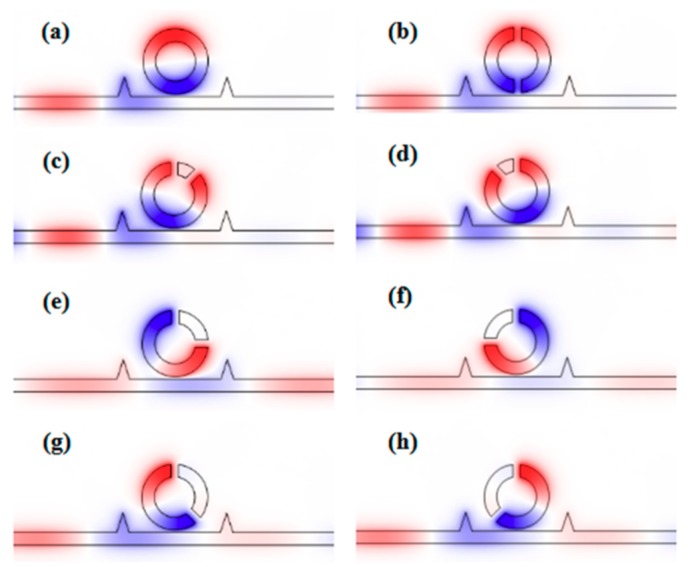
The H_Z_ field distribution at the resonance dip of (**a**) the complete ring structure at *λ* = 935 nm; (**b**) *φ* = 180° side-coupled structure at *λ* = 910 nm; (**c**) *φ* = 45° side-coupled structure at *λ* = 830 nm; (**d**) *φ* = 315° side-coupled structure at *λ* = 830 nm; (**e**) *φ* = 90° side-coupled structure at *λ* = 1385 nm; (**f**) *φ* = 270° side-coupled structure at *λ* = 1385 nm; (**g**) *φ* = 135° side-coupled structure at *λ* = 1185 nm; (**h**) *φ* = 225° side-coupled structure at *λ* = 1185 nm.

Their normalized H_Z_ field was also investigated, as depicted in Figure 5. Three phenomena are observable in the figure: Firstly, there were two nodes in the complete ring structure, *φ* = 45°, *φ* = 180° and *φ* = 315° side-coupled CSRRC structures, whereas there was only one node in the other side-coupled CSRRC structures. Secondly, the filed distribution of the complete ring structure was symmetric about the reference line. The filed distribution of the *φ* = 45° and *φ* = 315° side-coupled CSRRC structures was symmetrical about the axis that was in the center between the two splits. However, the filed distribution of the others was asymmetric about the axis that was in the center between two splits. Thirdly, when the resonance occurred in the CSRRC, there were no SPPs in the short part of the CSRRC. It can be observed that the filed distribution of the *φ* = 45° and *φ* = 315° side-coupled CSRRC structure, the *φ* = 90° and *φ* = 270° side-coupled CSRRC structure, and the *φ* = 135° and *φ* = 225° side-coupled CSRRC structure was similar. These three phenomena occurred because the different positioning of the splits would lead to different field distribution in the CSRRC. Therefore, it is necessary to choose a better position to make splits. According to the above analysis, the *φ* = 135° and *φ* = 225° side-coupled CSRRC structures have ultra-low transmittance at the dip, a relatively narrow FWHM and fewer detection interference factors. Hence, we chose to study one of them—the *φ* = 135° side-coupled CSRRC structure—in the rest of this paper.

**Figure 6:**
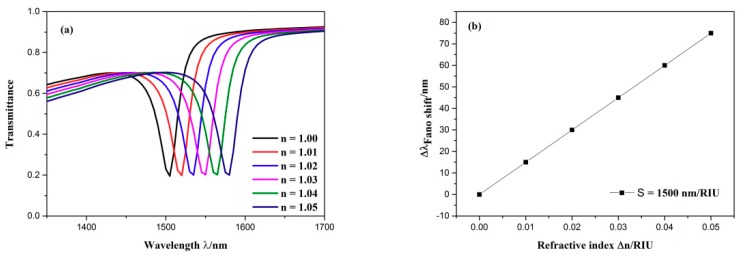
(**a**) Transmission spectra for diverse refractive indices. (**b**) the change of the dip wavelength with the variation of refractive index.

When the refractive indices of the dielectric change, the position of the Fano resonance will move with it. Accordingly, the resonance wavelength that we can detect will change. So different refractive indices can be attained by detecting different resonance wavelengths. Furthermore, the transmittance spectrum of Fano resonance is sharp so that it is sensitive to the change of refractive indices. Therefore, the influence of diverse refractive indices on the sensing properties of the designed structure was studied. The structural parameters were as follows: *R* = 160 nm, *l* = 30 nm, *φ* = 135°, *H* = 280 nm, *h* = 120 nm, *g* = 10 nm. The refractive index was gradually set as 1.00, 1.01, 1.02, 1.03, 1.04, 1.05 RIU. It was then found, as shown in Figure 6a, that as the refractive index increased, the transmission spectra exhibited an obvious redshift. The sensitivity (S) and the figure of merit (FOM) are two crucial parameters for weighting the capability of the sensing system. One can be expressed by S = ∆*λ*/∆n, where the change of resonance wavelength is described by ∆*λ* and the variation of refractive index is expressed by ∆n, and the other can be defined as FOM = S/FWHM [39]. Here, it is necessary to explain that there are two methods for defining the FOM, and the value of the FOM we get using this method is much lower than in that of the other method.

As shown in Figure 6b, when the variation of refractive indices changed, the wavelength shift of the dip altered linearly. As a result, this structure can serve as a refractive index sensor because it has the advantage of being more easily detected. The sensitivity of the sensor was obtained by linear fitting, which was 1500 nm/RIU with a FOM of 62.5. This is the best parameter for this structure.

From the above analysis, it is found that Fano resonance was formed by the interference between the waveguide with two symmetric triangle stubs and the CSRRC. It can be inferred from this that the propagation characteristics of the designed sensor are affected by changing its geometric parameters. Hence, it is essential to discuss the effects of different geometric parameters on propagation properties. In the following analysis, the default geometry parameter values are the same as in Figure 3.

**Figure 7:**
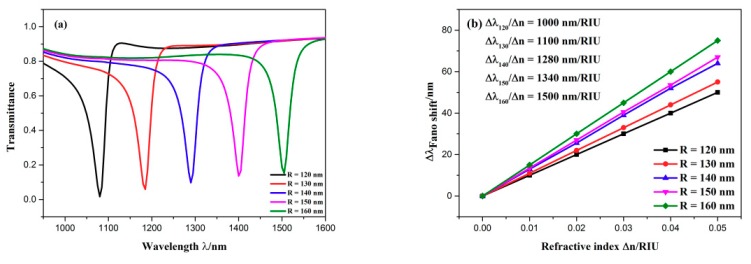
(**a**) Transmission spectra of the sensing system for diverse CSRRC outer radii; (**b**) the change of the dip wavelength with the variation of refractive index.

Firstly, the effects of the CSRRC outer radius on Fano resonance are discussed. The CSRRC outer radius *R* was adjusted from 120 to 160 nm for an interval of 10 nm, while other parameters remained unaltered. The transmission spectra are plotted in Figure 7a. As *R* increased, the dip position of Fano resonance showed an obvious redshift and the transmittance at the dip became a little higher. The simulation result indicates that the dip wavelength of Fano resonance was determined by *R*, which is a key parameter of the CSRRC. In other words, the wavelength of the dip relies on the CSRRC corresponding to the discrete narrowband state. By linear fitting, the different sensitivities of the various structures were obtained, as is shown in Figure 7b. It was found that sensitivity became better with the increase of the CSRRC outer radius *R.* Thus, it is necessary to make a compromise among device size, transmittance and sensitivity.

**Figure 8:**
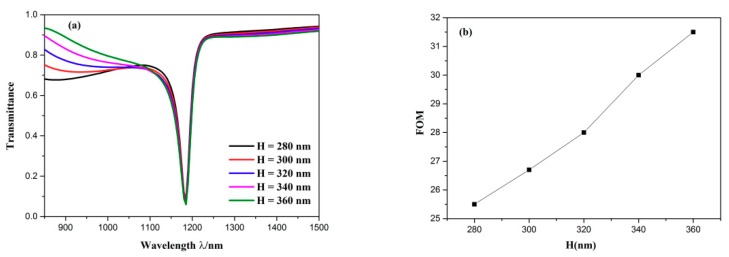
(**a**) Transmission spectra for the diverse distance between the two symmetric triangle stubs; (**b**) variation of FOM with the increase of distance between the two symmetric triangle stubs.

Successively, the influence of the distance between the two symmetrical triangle stubs *H* on the propagation characteristics was investigated. The distance between the two symmetric triangle stubs *H* was adjusted from 280 to 360 nm with intervals of 20 nm, while the other parameters remained invariable. As plotted in Figure 8a, the dip position of Fano resonance remained unchanged regardless of how *H* changed. When *H* increased, the transmittance of the dip slightly decreased and the FWHM of the transmission spectra became broader, which is displayed in Figure 8b. When the FWHM becomes smaller, a higher FOM and a better sensing resolution can be obtained. Thus, a smaller *H* should be applied to our sensor system. The distance between the two symmetric triangle stubs *H* is one of the significant parameters of the waveguide with two symmetric triangle stubs, which is considered as the continuous broadband state. Hence, it can be inferred from these calculation results that the continuous broadband state affects the lineshape of Fano resonance rather than its wavelength of the dip.

**Figure 9:**
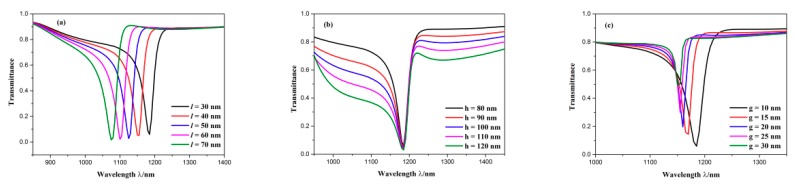
Transmission spectra for (**a**) diverse lengths of the CSRRC split; (**b**) diverse heights of triangle stub; (**c**) diverse coupling distances.

To further study the influence of geometric parameters on transmission properties, we altered the length of the CSRRC splits *l*. The *l* was adjusted from 30 to 70 nm with intervals of 10 nm, and here, other parameters were kept constant. Their transmission spectra can be observed from Figure 9a. The dip position of Fano resonance produced a blueshift and the Fano lineshape remained unchanged as the length of the CSRRC splits *l* increased. Then, we kept other parameters constant except for increasing the height of triangle stub *h* from 80 to 120 nm, with intervals of 10 nm. The simulation results of the different heights of the triangle stubs are depicted in Figure 9b. It is found that the dip wavelength of Fano resonance did not shift and the Fano lineshape changed from a nearly symmetrical shape to a completely asymmetrical shape, while increasing the height of triangle stub *h*. Next, we increased the coupling distance from 10 to 30 nm with intervals of 5 nm and kept other parameters invariable. The transmission characteristics can be attained from Figure 9c. When the coupling distance *g* became larger, the Fano resonance dip showed a blueshift, the FWHM got narrower, and the transmittance of the Fano resonance dip became higher. This can be explained by the fact that the coupling strength becomes weaker with the increase of the coupling distance *g*. Based on the above analysis, it can be concluded that the parameters of the CSRRC can change the dip wavelength of Fano resonance, whereas the parameters of the waveguide with two symmetric triangle stubs can change the shape of transmittance spectrum. This is attributed to the stronger field energy restriction of the ring cavity rather than the waveguide, which is displayed in Figure 3b.

## 4. Conclusions

In this paper, a compact refractive index sensor was theoretically presented, comprising a MIM waveguide with two symmetric triangle stubs coupled with a circular split-ring resonance cavity (CSRRC). Its transmission properties were studied by using the finite element method. Calculation results revealed that a Fano resonance emerged in the transmission spectrum, which was aroused by interference between the continuous broadband state related to the waveguide with two symmetric triangle stubs and the discrete narrowband state related to the CSRRC. By comparing it with similar structures, it has been found that the *φ* = 135° side coupled CSRRC structure that was studied in this paper has superior properties. In addition, the Fano resonance is significantly determined by the structural parameters of the sensing system. The dip wavelength of Fano resonance largely depends on the geometric parameters of the CSRRC, i.e., *R* and *l*. The lineshape of the transmission spectra are susceptible to the structural parameters of the waveguide with symmetric two triangle stubs, i.e., *H* and *h*. Particularly, the coupling distance influences both the dip wavelength of the Fano resonance and the spectra lineshape. The sensitivity of the designed system can reach 1500 nm/RIU with a high FOM of 65.2. The proposed structure is highly promising for nanophotonic applications.
